# Socioeconomic and risk-related drivers of compliance with measures to prevent SARS-CoV-2 infection: evidence from the Munich-based KoCo19 study

**DOI:** 10.1186/s12889-023-15759-9

**Published:** 2023-05-11

**Authors:** Sara Pedron, Michael Laxy, Katja Radon, Ronan Le Gleut, Noemi Castelletti, Jessica Michelle Guggenbüehl Noller, Maximilian Nikolaus Diefenbach, Michael Hölscher, Reiner Leidl, Lars Schwettmann, the KoCo19 collaboration group, Felix Forster, Abhishek Bakuli, Judith Eckstein, Günter Froeschl, Otto Geisenberger, Christof Geldmacher, Arlett Heiber, Larissa Hoffmann, Kristina Huber, Dafni Metaxa, Michel Pletschette, Camilla Rothe, Mirjam Schunk, Claudia Wallrauch, Thorbjörn Zimmer, Michael Pritsch, Andreas Wieser, Laura Olbrich, Verena Thiel, Friedrich Riess, Inge Kroidl, Elmar Saathoff, Stephan Prückner, Eleftheria Zeggini, Christiane Fuchs, Jan Hasenauer, Fabian Theis

**Affiliations:** 1 Professorship of Public Health and Prevention, Technical University of Munich, Munich, Germany; 2 Institute of Health Economics and Health Care Management, Helmholtz Zentrum München, German Research Center for Environmental Health (GmbH), Neuherberg, Germany; 3 German Center for Diabetes Research (DZD), Neuherberg, Germany; 4 Global Diabetes Research Center, Rollins School of Public Health, Emory University, Atlanta, GA USA; 5 Department of Sport and Health Sciences, Technical University of Munich, Munich, Germany; 6 Center for International Health, Institute for Occupational, Social and Environmental Medicine, University Hospital, Ludwig Maximilian University, Munich, Germany; 7 Core Facility Statistical Consulting, Helmholtz Zentrum München, German Research Center for Environmental Health (GmbH), Munich, Germany; 8 Division of Infectious Diseases and Tropical Medicine, University Hospital, Ludwig Maximilian University, Munich, Germany; 9 Institute of Radiation Medicine, Helmholtz Zentrum München, German Research Center for Environmental Health (GmbH), Neuherberg, Germany; 10 Center for International Health, University Hospital, Ludwig Maximilian University, Munich, Germany; 11 German Center for Infection Research (DZIF), Partner Site Munich, Munich, Germany; 12 Munich School of Management and Munich Center of Health Sciences, Ludwig Maximilian University, Munich, Germany; 13 Department of Health Services Research, School of Medicine and Health Sciences, Carl Von Ossietzky University of Oldenburg, Oldenburg, Germany

**Keywords:** SARS-CoV-2, Covid-19, Compliance, Non-pharmaceutical interventions, Regulations, Measures

## Abstract

**Objectives:**

Although a growing share of the population in many countries has been vaccinated against the SARS-CoV-2 virus to different degrees, social distancing and hygienic non-pharmaceutical interventions still play a substantial role in containing the pandemic. The goal of this study was to investigate which factors are correlated with a higher compliance with these regulations in the context of a cohort study in the city of Munich, southern Germany, during the summer of 2020, i.e. after the first lockdown phase.

**Methods:**

Using self-reported compliance with six regulations and personal hygiene rules (washing hands, avoiding touching face, wearing a mask, keeping distance, avoiding social gatherings, avoiding public spaces) we extracted two compliance factor scores, namely *compliance with personal hygiene measures* and *compliance with social distancing regulations*. Using linear and logistic regressions, we estimated the correlation of several socio-demographic and risk perception variables with both compliance scores.

**Results:**

Risk aversion proved to be a consistent and significant driver of compliance across all compliance behaviors. Furthermore, being female, being retired and having a migration background were positively associated with compliance with personal hygiene regulations, whereas older age was related with a higher compliance with social distancing regulations. Generally, socioeconomic characteristics were not related with compliance, except for education, which was negatively related with compliance with personal hygiene measures.

**Conclusions:**

Our results suggest that for a targeted approach to improve compliance with measures to prevent SARS-CoV-2 infection, special attention should be given to younger, male and risk-prone individuals.

**Supplementary Information:**

The online version contains supplementary material available at 10.1186/s12889-023-15759-9.

## Introduction

By the time this paper was written, in quite a number of countries, large parts of the population have been vaccinated against the Sars-CoV-2 virus [1]. Yet, protection is varying by type and number of vaccinations received, and individual risk [2]. With risks remaining, but also due to vaccine hesitancy, new variants of concern, and restricted access to vaccines in the Global South [3], achieving global herd immunity remains a target hard to achieve or not even feasible at all. Therefore, curbing the spread of SARS-CoV-2 infection still depends on non-pharmaceutical interventions (NPIs), including those targeting individual behavior. Specific guidelines issued by local and national authorities for mask wearing, maintaining distance and maximum number of individuals allowed in stores and other closed rooms serve as the most prominent public health measures. Other suggestions include washing hands frequently and thoroughly, avoiding touching one’s face, avoiding open, crowded spaces and gatherings [4–7].

The efficacy of these behaviors in preventing the spread of the SARS-CoV-2 virus depends on the type of measure [8–10], but also on the degree and constancy to which a society follows them. Furthermore, the ongoing scientific debate and evidence have highlighted that transmission of the virus is mostly airborne, so that measures, such as mask wearing and avoiding closed crowded spaces, have risen as highly relevant behaviors to limit transmission [11–13]. Therefore, understanding compliance with guidelines and its determinants is important to derive evidence-based strategies to increase it, to inform modelling of disease spread, and to help managing future infectious diseases or even pandemic situations.

Previous studies have shown that personal beliefs regarding perceived vulnerability and severity of the disease, and efficacy of the measures, are among the most important drivers of compliance [14–26]. Furthermore, some studies highlighted that actual risk and cues to action, such as government recommendations, have a positive effect on compliance [27, 28], whereas Xu and Cheng [29] showed no effect.

Furthermore, individual demographic characteristics play an important role. In fact, being older, being a woman, being married, having a migration background and the number of children in the household are usually associated with higher compliance [15, 16, 23, 30–33]. Furthermore, the number of comorbidities was shown to be associated with higher compliance [17], while having had Covid-19 symptoms was not related with compliance [16]. The role of socioeconomic status (SES) has shown mixed effects. Some studies indicated that a higher SES (higher level of education, higher income, being employed) is related with lower compliance [16, 17, 30, 32]. However, other studies showed that lower regional deprivation and development levels are related with lower compliance [22, 23, 27], while Lieberoth et al. [18] did not confirm this compliance pattern.

Individual psychosocial characteristics are also relevant, but evidence is scarce. Personality traits, altruism, social responsibility, trust in government or science, patience and perceived behavioral control are usually related to higher compliance, with some exceptions [14–18, 20, 30, 32–44]. Regarding economic preferences, the evidence for the role of time preference is controversial. Some authors found no effect [17, 35] whereas Byrne et al. [45] found an association between greater temporal discounting and compliance. Regarding risk preferences, which we consider as predictors in our work, most studies showed that risk aversion is positively related with compliance [17, 29, 33, 35, 45–47], while other authors evidenced no effect of this factor [41].

In this study, we provide further evidence regarding the correlates of compliance with NPIs by analyzing a rich set of data collected between June and December 2020 in the city of Munich (Bavaria) [48]. Based on a large set of potential determinants and confounders, including socioe-conomic characteristics, living conditions, individual risk attitudes and perceptions, actual infection risk, existing comorbidities, being employed in a risky job, and previous SARS-CoV-2 infection, we aimed at testing which factors are associated with compliance behavior. The analyzed data represent thus an important, rather extensive and unique source of information on compliance behavior during the first stages of the pandemic, in a time when most measures were softened after the first lockdown, while pandemic fatigue was still low [49] and compliance was mostly based on individual behavioral decisions rather than strict measures in place.

## Methods

### Data

We analyzed data from the KoCo19 study (*prospective community-based Covid-19 cohort*) based in the city of Munich, Bavaria, south Germany. For the study, 2,994 households were selected by random walk door-to-door methodology within regional clusters in the city of Munich. All household members older than 14 years of age were eligible. A detailed description can be found in the study protocol [48]. All methods were carried out in accordance with relevant guidelines and regulations.

**Fig. 1:**
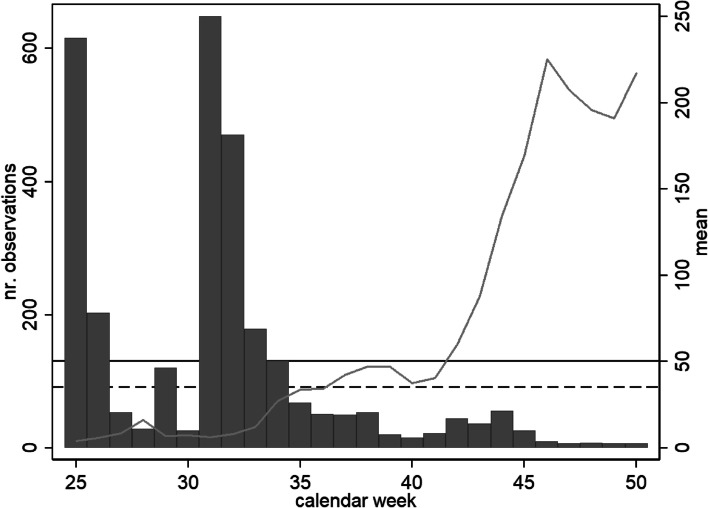
Number of observations and weekly mean of 7-day cumulative incidence per 100,000 individuals within the follow-up period considered (Landeshauptstadt München, 2021). Horizontal lines indicate the two thresholds that were introduced from authorities to indicate a sharpening of contact restrictions (35 – dashed line, and 50 – solid line)

Data collection at baseline, which included collection of blood samples and questionnaire information, took place between 6^th^ of April 2020 and 12^th^ of June 2020. A follow-up investigation was carried out by post, involving the collection of questionnaire data and blood samples [50]. Responses reached the study center between 19^th^ of June 2020 and 10^th^ of December 2020, whereby 90% of responses were received before mid-September 2020 (calendar week 37, see Fig. 1). The questionnaires at baseline focused on gathering socioeconomic, demographic and health status information of the respondents, whereas the questionnaire at follow-up focused mainly at collecting information on present compliance with the public health measures, risk perception, risk aversion, self-efficacy and behavioral information.

We utilized both baseline (sociodemographic and health status) and follow-up (compliance, risk perception, risk aversion and self-efficacy) questionnaire information. Of the 5,313 individuals surveyed at baseline, 2,995 individuals (from 1,926 households, i.e., on average 1.5 individuals per household) responded to the follow-up investigation. Furthermore, since we were explicitly interested in investigating the association of socioeconomic status including completed educational level and employment status with compliance, we decided to focus on the adult population (*N* = 2,880), excluding children and adolescents younger than 20 years of age (*N* = 115).

These data offer a unique opportunity to analyze compliance behavior during a period of the Covid-19 pandemic, in which no hard lockdown was in place, but containment was dependent on a series of measures and recommendations focusing on individual behavior. In fact, starting from June 2020, the hard confinement measures imposed during the first lockdown were eased in Germany. Mask wearing had been mandatory in supermarkets, public transport and some public spaces, while it was generally recommended everywhere else since the end of April 2020 [51]. Restrictions on social contacts and travels depended on the infection rates. Cutoffs based on the number of incident cases were employed starting from June and July 2020 to signalize a change in restrictions [52, 53]. In all cases, individuals were suggested and encouraged to always maintain 1.5 m distance and to wash hands frequently. Therefore, our analysis might help to shed light on the determinants of compliance in phases when individual behaviors play a central role.

### Variables

#### Outcomes

In the follow-up questionnaire, participants were asked how regularly they adhered with a series of six preventive hygiene and social distancing measures in the two previous weeks. A similar way of measuring compliance with Covid-19 measured has been used in previous studies [15, 54]. The measures included washing hands for 20 s, touching eyes, nose, and mouth only with clean hands, wearing a face mask, keeping a distance of 1.5 m in public, avoiding private social gatherings, and avoiding public spaces. Responses were given on a 5-point Likert scale, from “never” to “always” (in between “seldom”, “sometimes”, and “often”). To investigate the dimensionality of compliance we carried out an exploratory factor analysis. Since the potential underlying factors are likely to be correlated, we computed rotated factor loadings with a promax rotation [55]. We then extracted the two resulting factor scores of compliance and used them as continuous outcome variables in the main analysis. These scores were related to (1) compliance with personal hygiene regulations and (2) compliance with social distancing measures. A full description is given in the results section.

As a supplementary analysis, we analyzed the single compliance behaviors as dichotomous variables. Individuals who reported that they followed the measures at least “often” were considered compliant. We tested alternative definitions of this dichotomization in the sensitivity analyses.

#### Correlates of compliance

As described in the literature, the potential predictors of compliance are manifold, including individual sociodemographic characteristics, situational factors (actual/communicated risk of infection, seasonality, mode of implementation of the measures), personality traits and preferences, personal trust in government/science, perceived benefits from the measures etc. Due to data availability, we were able to consider only a selection of these factors.

We distinguished three classes of factors that might influence compliance with preventive measures. First, we incorporated individual socioeconomic and demographic characteristics measured at baseline, including age, sex, migration background, education, employment status, monthly household income, housing type, living conditions, living with a partner, number of children in the household and working in risky jobs (i.e. working in the health sector or working in another essential sector with public contact, such as public transport). Furthermore, we included information on the individual health status, such as the presence of chronic conditions or of a positive serologic test for SARS-CoV-2 at baseline. All details on these variables are reported in Supplementary material 1.

Second, we included information on the actual risk of infection based on the 7-day cumulative Covid-19 incidence per 100,000 individuals as reported by official statistics of the city of Munich [56]. For the analysis, we considered the highest value of the cumulative 7-day incidence per 100,000 inhabitants in the seven days prior to reception of the postal follow-up questionnaire. We categorized these data based on the official cutoffs that were used by local health authorities as “cues to action”, to signal the risk of infection and to introduce new regulations (0–35, >35–50, > 50) [52, 53].

Third, we investigated the correlation between a series of factors on risk perception, risk aversion and self-efficacy, measured at follow-up, with compliance. We also included subjective infection risk, illness severity and self-reported subjective level of information (i.e. how well informed a person feels regarding the situation), all assessed on a 7-point Likert scale. Furthermore, using factor analysis, we generated a factor score on “risk perception” based on three variables assessing personal perception on closeness, spreading speed and gravity of the disease on a 7-point Likert scale. Additionally, again using factor analysis, we generated a score on the dimension “worry”, based on the self-reported individual concerns regarding losing someone close, the own finances, losing one´s job as well as the general economic situation. The extent to which these situations worried individuals was measured on a 7-point Likert scale. We report detailed information on both scales (“risk perception” and “worry”) in Supplementary material 2. Risk aversion in the health domain was measured by a 10-point Likert scale, from (1) “not at all ready to take risks” to (10) “very likely to take risks” [57]. In contrast to the original 11-point scale, the scale used in the present study does not allow responders to report indifference, i.e. answers at the mid-point of their scale. Self-efficacy was measured by reverse-coding agreement to the statement “The new Coronavirus is something against which: (1) I can actively do something to (7) I feel completely helpless”.

Lastly, we controlled for seasonality (summer/fall), based on the day when the postal follow-up was received in the study center.

Continuous variables (subjective infection risk, subjective infection severity, level of information, risk-aversion and self-efficacy) were standardized (mean equal to zero, standard deviation equal to one) for ease of interpretation.

### Statistical analysis

Due to some missing values in the considered predictors of compliance, we carried out multiple imputation using predictive mean matching with thirty replications for all determinants included in the model. Full descriptive statistics and imputation diagnostics are available in Supplementary material 3.

To estimate the effect of the determinants on the compliance scores, we carried out a linear regression based on all 30 imputation replications. Furthermore, to estimate the effect of the determinants on the six behaviors, we carried out logistic regressions using the dichotomized behaviors as outcomes and reported effect estimates as Odds Ratios (OR), again, based on imputed data.

Additionally, we carried out several robustness checks. First, we tested our analysis on the original data without imputation. Second, we carried out the analysis excluding individuals with a positive serologic test at baseline (*N* = 46), because of potential interaction effects with the considered correlates of compliance. Third, because in our dataset the incidence rates (“actual risk of infection”) are highly correlated with the season (Chi-square *p*-val < 0.001, Cramer´s V = 0.90), we explored different models including these factors separately. No other collinearity issues were detected using the variance inflation factor (VIF).

As we considered multiple potential correlates of compliance, our results might present problems of multiple testing, potentially leading to type 1 errors. Therefore, all p-values were corrected for the number of independent variables within a regression using the Benjamini & Hochberg method [58]. All analyses were performed using STATA 14 (College Station, Texas) and R (Version 4.0.3).

## Results

### Descriptive statistics

Our study population included 54% females (Table 1). 60% of the sample was between 35 and 64 years of age. Among the 60% employed, 9% were employed in the health sector, whereas 18% reported to be employed in a risky job outside the health sector.

**Table 1:** Descriptive statistics of sociodemographic, health status, actual infection risk and seasonality covariates

	**N**	**Freq.**	**Mean (SD)**
**Sociodemographic**			
Age group			
20–34	636	22%	
35–49	838	29%	
50–64	852	30%	
65–79	440	15%	
80 +	114	4%	
Sex			
Female	1567	54%	
Male	1313	46%	
Education Level			
Low (< 12 years of school)	910	32%	
High (≥ 12 years of school)	1970	68%	
Employment status			
Employed	1730	60%	
Self-employed	265	9%	
Retired	592	21%	
Not employed	293	10%	
In risky job (health)			
Yes	203	7%	
No	2677	93%	
In risky job (other)			
Yes	429	15%	
No	2451	85%	
Monthly HH income (€)			
≤ 2500	488	17%	
2500–4000	644	22%	
>4000–6000	951	33%	
> 6000	797	28%	
Housing type			
1–4 apartments	983	34%	
≥ 5 apartments	1897	66%	
Living area per individual			
≤ 40sqm/individual	1604	56%	
> 40sqm/individual	1276	44%	
Living with partner			
Yes	2363	82%	
No	517	18%	
Number of children			0.63 (0.96)
Born outside Germany			
Yes	434	15%	
No	2446	85%	
**Health status**			
Any chronic illness			
Yes	1240	43%	
No	1640	57%	
Positive serologic test at BL			
Yes	47	2%	
No	2833	98%	
**Actual infection risk**			
7 day incidence/100,000 inhabitants			
0–35	2525	88%	
> 35–50	173	6%	
> 50	182	6%	
**Season**			
Summer	2664	93%	
Fall	216	8%	

Summaries from the first imputation, only variables with missing data were imputed

For more information, see Supplementary material 3

*HH* Household, *BL* Baseline

The descriptive statistics of the risk perception variables and the constructed scores are reported in Table 2. On average, included individuals reported high risk aversion levels and self-efficacy levels.

**Table 2:** Descriptive statistics of risk perception covariates

	**Total**	**Mean (SD)**
**Risk perception**		
Subjective infection risk	2880	3.42 (1.26)
Subjective infection severity	2880	4.19 (1.42)
Level of information	2880	5.09 (1.16)
* (Score: risk perception)*	2880	0.00 (1.00)
Risk (near)	2864	4.56 (1.42)
Risk (quick)	2871	4.74 (1.53)
Risk (threat)	2870	5.16 (1.49)
*(Score: worry)*	2880	0.00 (0.99)
Worry (lose someone)	2873	4.06 (1.87)
Worry (own economic situation)	2872	3.14 (1.82)
Worry (economy)	2870	5.10 (1.50)
Worry (job)	2258	2.75 (1.87)
Risk aversion	2880	7.18 (2.10)
Self-efficacy	2880	4.64 (1.63)

Summaries from the first imputation, only variables with missing data were imputed; the factor scores, not the original values, were imputed for risk perception and worry scores. All variables were standardized for the analysis

For more information, see Supplementary material 3

### Exploratory and confirmatory factor analysis of compliance

Overall, most individuals reported a high compliance with the regulations (Table 3). The most accepted behavior was wearing a mask (96%), followed by keeping 1.5 m distance in public (89%) and washing hands for 20 s (83%). The recommendations to avoid touching the face (71%), avoid social gatherings (65%) and public spaces (65%) were less frequently followed.

**Table 3:** Frequencies and descriptive statistics of the outcomes

			**Compliant**		
	**N**	**Mean (SD)**	**Yes**	**No**	**Freq.**
**Washing hands**	2,872	4.22 (0.88)	2,394	478	83%
**Avoiding touching face**	2,874	3.79 (0.98)	2,033	841	71%
**Wearing a mask**	2,870	4.58 (0.63)	2,743	127	96%
**Keeping 1.5 m distance**	2,873	4.15 (0.66)	2,564	309	89%
**Avoiding social gatherings**	2,868	3.72 (1.41)	1,854	1,014	65%
**Avoiding crowded public spaces**	2,870	3.62 (1.00)	1,855	1,015	65%

"compliant: yes": N and share of individuals who followed at least often the investigated recommendations

*SD* Standard deviation

The exploratory factor analysis revealed the presence of two underlying factors (Table 4). By allowing the underlying factors to be correlated (correlation coefficient = 0.31), the behaviors loading onto each factor could be identified. The first factor was composed of the underlying behaviors “washing hands”, “avoid touching face” and “wearing mask”. Because these behaviors concern personal hygiene, we termed the factor henceforth “personal hygiene” factor. The second factor gathered the remaining behaviors, i.e. “keeping 1.5 m distance”, “avoiding social events” and “avoiding public spaces”; therefore, we termed it “social distancing” factor.

**Table 4:** Results of the factor analyses

	**Unrotated factor loadings**		**Rotated factor loadings**			
	**Factor 1**	**Factor 2**	**Factor 1**	**Factor 2**	**Alpha**	**Alpha new**
**Factor 1: personal hygiene**					0.597	
Washing hands	**0.648**	**-0.447**	**0.800**	-0.045		0.408
Avoiding touching face	**0.650**	**-0.423**	**0.782**	-0.022		0.431
Wearing a mask	**0.581**	-0.260	**0.604**	0.086		0.598
**Factor 2: social distancing**					0.540	
Keeping 1.5 m distance	**0.640**	0.144	0.322	**0.480**		0.524
Avoiding social gatherings	**0.514**	**0.614**	-0.140	**0.833**		0.503
Avoiding crowded public spaces	**0.663**	**0.464**	0.082	**0.780**		0.272
Eigenvalue	2.294	1.236	1.957	1.773		
% Shared variance accounted for	0.38	0.18	0.33	0.30		
Correlation between factors			0.310			
Observations	2,834					

In bold, factor loadings whose absolute value is higher than 0.4; alpha: Cronbach’s alpha; alpha new: new Cronbach´s alpha value if the item is deleted

Both factors had a satisfactory Cronbach´s alpha level (personal hygiene = 0.597, social distancing = 0.540). The Cronbach’s alpha could not be improved by eliminating any of the underlying behaviors. We coded both factors so that higher values indicate more compliant behavior. More details on the distribution of the final scales are available in Supplementary material 2.

### Variables associated with compliance

The variables associated with the compliance scores differed depending on the outcome considered (Table 5). For the compliance score “personal hygiene”, low education, being retired and being born outside Germany were associated with higher self-reported compliance. On the contrary, being male was related with a lower compliance with personal hygiene measures. Furthermore, socioeconomic status (i.e., income level, employment status) was not associated with compliance with personal hygiene measures. With respect to risk perception, the level of information, perceived severity of SARS-CoV-2 infection and personal risk perception score were associated with higher compliance with personal hygiene measures. Furthermore, both risk aversion and self-efficacy were associated with a higher compliance with personal hygiene recommendations. Specifically, risk aversion was the variable that had the higest correlation with compliance in both domains among the risk perception variables, amounting to 0.21 points increase in compliance for 1 SD increase in risk aversion for the personal hygiene dimension and 0.18 points for the social distancing dimension.

**Table 5:** Analysis results for the two compliance scores

	**Compliance—personal hygiene**			**Compliance—social distancing**		
	**OLS est**	**95% CI**	**Corrected pval**^**#**^	**OLS est**	**95% CI**	**Corrected pval**^**#**^
***Sociodemographic***						
**Age**	-0.001	[-0.036; 0.034]		0.079	[0.043; 0.115]	***
**Male**	-0.326	[-0.392; -0.261]	***	-0.045	[-0.107; 0.018]	
**Low education (vs. High)**	0.162	[0.072; 0.252]	***	0.028	[-0.059; 0.116]	
**Employment Status (ref: employed)**						
**Self Employed**	-0.031	[-0.152; 0.091]		-0.063	[-0.188; 0.062]	
**Retired**	0.242	[0.108; 0.375]	***	0.152	[0.025; 0.279]	
**Others-not employed**	0.116	[-0.002; 0.234]		0.044	[-0.08; 0.168]	
**In risky job (health)**	0.049	[-0.093; 0.191]		-0.004	[-0.148; 0.139]	
**In risky job (other)**	-0.031	[-0.133; 0.07]		-0.111	[-0.225; 0.003]	
**Living with partner**	-0.045	[-0.178; 0.087]		0.012	[-0.119; 0.143]	
**Number of children**	-0.014	[-0.062; 0.034]		0.025	[-0.023; 0.073]	
**Monthly HH income (ref: ≤ 2500 €)**						
**2500- ≤ 4000**	-0.003	[-0.144; 0.138]		-0.065	[-0.208; 0.078]	
**4000- ≤ 6000**	0.001	[-0.145; 0.148]		-0.045	[-0.191; 0.100]	
**> 6000**	0.020	[-0.141; 0.181]		-0.095	[-0.259; 0.068]	
**Housing type (≥ 5 apts)**	0.001	[-0.083; 0.085]		-0.032	[-0.116; 0.053]	
**Living area (> 40sqm/ind)**	-0.062	[-0.16; 0.036]		0.016	[-0.083; 0.114]	
**Born outside Germany**	0.166	[0.064; 0.267]	**	0.078	[-0.023; 0.180]	
***Health status***						
**Any chronic illness**	0.063	[-0.012; 0.138]		0.001	[-0.075; 0.076]	
**Positive serologic test (BL)**	-0.408	[-0.717; -0.100]	*	-0.378	[-0.655; -0.102]	*
***Actual infection risk (ref: 0–35)***						
**> 35–50**	-0.027	[-0.192; 0.138]		0.210	[0.055; 0.364]	*
**> 50**	0.016	[-0.256; 0.288]		0.369	[0.017; 0.720]	
***Risk perception***						
**Subjective infection risk (SD)**	-0.012	[-0.051; 0.027]		-0.072	[-0.112; -0.033]	***
**Level of information (SD)**	0.096	[0.057; 0.135]	***	0.048	[0.01; 0.086]	*
**Subjective infection severity (SD)**	0.060	[0.018; 0.101]	*	0.055	[0.012; 0.098]	*
**Risk perception (score)**	0.130	[0.087; 0.173]	***	0.156	[0.112; 0.201]	***
**Worry (score)**	0.046	[0.003; 0.088]		0.032	[-0.011; 0.074]	
**Risk aversion (SD)**	0.209	[0.169; 0.249]	***	0.179	[0.140; 0.219]	***
**Self-efficacy (SD)**	0.061	[0.023; 0.100]	**	0.037	[-0.001; 0.076]	
**Season (Fall) (ref: Summer)**	0.233	[-0.017; 0.484]		0.184	[-0.146; 0.514]	
**Intercept**	0.049	[-0.167; 0.265]	***	-0.375	[-0.597; -0.152]	**
**N**	2858			2854		
**R**^**2**^	0.202			0.204		

*HH* Household, *BL* Baseline, *SD* Standard deviation; Since we did not impute the outcome variables (very few missing values), the respective models only include a slightly reduced subset of observations

^#^corrected pval: significance of the *p*-value corrected using the Benjamini & Hochberg method. Significance: *p < .05, **p < .01, ***p < 0.001

Compliance with social distancing measures increased with increasing age, while no other sociodemographic variable included showed a significant correlation. Among the risk perception correlates, subjective infection severity, the risk perception score and individual risk aversion were positively related with compliance. Notably, for this outcome, subjective infection risk was negatively associated with compliance with social distancing regulations.

### Correlates of the single behaviors

The analysis for the single behaviors revealed slightly different mechanisms depending on the single behavior considered (Supplementary material 4). A higher age category was related with a lower frequency of wearing a mask, but also with a higher frequency of keeping the 1.5 m distance and avoiding public spaces. Being male was positively and significantly associated with a lower adherence to washing hands and to the avoidance of touching one’s own face. The socioeconomic variables were not significantly associated with any of the behaviors. The only exception was a higher odd of wearing a mask for retired individuals. Having received a positive serologic test at baseline was related with a very low compliance to wearing a mask (OR = 0.24 [0.10 – 0.59]) and washing hands (OR = 0.37 [0.18 – 0.74]).

Among the risk perception variables, the risk perception score (including subjective perceptions of how near, quick and threatening the virus is) and risk aversion were consistently, positively and significantly associated with all behaviors considered.

### Sensitivity analysis

All sensitivity analyses confirmed the robustness of the main analysis (Supplementary material 5).

The robustness check considering separately the correlates of compliance “actual risk of infection” and “season” revealed that these two variables, considered separately, were positive and significantly related with compliance in both factors “personal hygiene” and “social distancing” (Supplementary material 5). This revealed an increase in compliance for both factors in fall compared to summer, but in our analysis, it was not possible to disentangle these effects.

## Discussion

In this study, we showed that compliance with regulation entails two underlying factors, one related to “personal hygiene” and the other related to “social distancing”. Furthermore, our analysis indicated that several demographic variables were related to higher compliance. Women, low-educated individuals, retired individuals and people born outside Germany reported a higher compliance with personal hygiene regulations, while older individuals stated a higher compliance with social distancing measures. Notably, the risk perception score and the personal risk aversion were significant and consistent determinants of the two latent factors and of all the single behaviors.

The identification of the two underlying factors reflects the results from other studies [14, 15, 17]. These studies identified similar compliance dimensions, such as “avoidant behavior” (which includes physical distancing, avoiding kissing and hugging with individuals outside of the close family) or “preventive behavior” (which includes hygienic precautions, such as washing hands and coughing into one’s sleeves). Although these indices are based on partly different behavioral measures (e.g. we could not include “coughing into ones sleeves” but we included “avoiding touching face”), we argue that the underlying latent factors of compliance can be compared across these studies. Regarding the underlying latent factors of compliance identified, one important distinction should be made in light of recent developments in the knowledge about the spreading mechanisms of the virus. At the time when the data were collected (summer/fall 2020), we still witnessed a debate whether the SARS-CoV-2 virus was mostly transmitted by contacts or smear infections or whether it was mostly airborne [59]. Further evidence confirmed that the virus is mostly airborne [11–13], which turned the focus towards containment measures on social distancing behaviors, whereas among the personal hygiene behaviors, especially mask wearing gained importance. This central point should be kept in mind while reading and interpreting the results of our study.

Regarding the correlates of compliance, the role of demographic characteristics is consistent with previous evidence [15–17, 23, 30–33]. Males were less likely than females to comply with personal hygiene regulations. Furthermore, the study shows that retired individuals follow regulations on personal hygiene more frequently than their employed counterparts. This might be related to the fact that retirees are less likely to have work or social obligations where following the regulations might be a difficult task. This result is also reflected in the effects on the age variable: older individuals tended to wear less often a mask, but they reported a higher compliance with social distancing regulations.

Additionally, in our sample, individuals with a migration background indicated that they followed personal hygiene regulations more frequently. This result also finds support in the previous literature [30]. The effect might be related to a different attitude towards the topic: different countries were hit with different intensity by the pandemic in the first months, with Germany surpassing the first wave of the pandemic with less victims [60]. These individuals might have perceived the pandemic and the gravity of the situation as more intense depending on their individual background and connections with their homeland and might also have been more affected by travel restrictions. Furthermore, as highlighted by other authors [30], this result is in line with the “immigrant health paradox”: individuals with a direct or indirect migration background tend to report better health behaviors and health outcomes than natives [61]. However, this result should be interpreted with caution, owing to the selected nature of the analyzed sample. Given that the questionnaire was available only in German, it is very likely that the sample of individuals with a migration background that were included in the study are individuals who know the German language well and are well integrated, thus systematically leaving out parts of the individuals with a recent migration background.

The result that socioeconomic characteristics are mostly not related with compliance add to the mixed results which can be found in the literature [16–18, 22, 23, 27, 30, 32]. These results confirm the fact that SES does not have an unequivocal role for compliance with Covid-19 regulations, unlike for compliance in other domains (e.g. with therapy recommendations), as previous studies have shown [62]. The only exception in our study is the higher compliance reported by low-educated individuals with personal hygiene recommendations. However, this result must be interpreted with caution due to the possible presence of social desirability bias in reporting subjective frequency of compliance with the considered behaviors. Further studies are needed to disentangle the effects behind this result, taking into account potential biases due to social desirability and health literacy.

Having had a positive serologic test at baseline, i.e., having had a Covid-19 infection before, is related with a lower compliance with wearing masks and washing hands. The awareness of a previous Covid-19 infection might have given individuals a higher degree of confidence to be immune to the illness. This behavior is problematic especially with the emergence of new variants of concern, with a potentially higher escape to immunity via infection from previous variants of the virus [63]. This result should be further investigated, but also interpreted with caution due to the low number of previously infected individuals in our sample. Furthermore, recent evidence showed that for individuals who were vaccinated and thus could expect a substantial protection against Covid-19, compliance with the regulations remains constant [33]. Further research should consider this difference as potential determinant of compliance with the regulation, since the two groups (those who experienced an infection and those who were vaccinated) are likely to be different with respect to unobserved confounders.

Furthermore, we investigated whether compliance with regulations changed in responses to higher levels of incidence and seasonality. The main analysis, which included both variables simultaneously, revealed no significant effect on compliance, probably because of the high correlation between these two variables. Higher incidence rates were positively related with compliance in the social distancing domain, albeit with a large confidence interval. Considering these two factors separately in the sensitivity analysis revealed that both were significantly related with a higher compliance with both personal hygiene and social distancing regulations. Thus, in our analysis we could observe an increase in compliance in fall, but we could not disentangle whether this increase was related with rising incidence rates or whether this was a seasonality effect. Therefore, the effect of these two factors should be further investigated, since they might represent important aspects to consider in policy intervention. In fact, the official cutoffs based on cumulative infection rates may represent important “cues to action”, which could have an impact on compliance rates. In light of recent changes in the communicated risk measures and related cutoffs in Germany, which directly take into account not only cumulative incidence rates but also hospitalization rates and capacity utilization of intensive care units [64], the changing effectiveness of these communication strategies should be analyzed in the future.

Within the risk perception category, several variables played an important role for compliance, making it one of the most important groups of correlated factors. Subjective level of information, subjective infection severity and the constructed risk perception score, which included speed, closeness and severity of the threat, are related with a higher compliance in both domains. This is in line with theoretical models of behavior, such as the Health Belief Model [65], in which threat perception and beliefs thereof are among the most important drivers of compliance. Previous empirical studies of the present pandemic have also sustained this finding [14–26]. Among this group of factors, we also obtained a counterintuitive result: a higher subjective infection risk was related with a lower compliance with social distancing measures. Since we are only estimating associations, the higher subjective infection risk might be interpreted as a direct result of the less frequent social distancing. In fact, individuals who are aware that they frequently engage in risky social behaviors (for example because of a risky employment that we missed in our analysis, of informal care or of personal choice), might also be aware that their infection risk is higher. Therefore, in this case, subjective infection risk would be a result of their compliance behavior, rather than a determinant.

Within the subjective risk perception category, the two psychosocial characteristics considered, namely risk aversion and self-efficacy, were also positively related with compliance. While a higher self-efficacy is especially related with avoiding touching one´s face and keeping distance, risk aversion is consistently and significantly related with each single and aggregated dimension of compliance and shows a very robust association across all our sensitivity analyses. This result confirms what was previously reported by other studies [17, 29, 33, 35, 45, 47, 66], though it contrasts findings of a study on college students [41]. Therefore, this result confirms risk aversion as one of the most important and consistent drivers of compliance with Covid-19 regulations. The fact that our result is based on a self-reported measure of risk aversion might cast doubt on this result. However, similar results were obtained using objective measures of risk aversion [17], offering a confirmation of our result. Nevertheless, identifying potential policy implications based on this result is challenging. As other authors have argued [35], specific groups of the population, for example identified by SES or occupational categories, might present higher levels of risk aversion and thus be targeted by specific interventions. The identification of such groups is however very context-specific: our results suggest that, even controlling for SES and occupational characteristics, risk aversion plays an independent role in our target population.

One strength of this study is the large number of factors included, covering several potential correlates of compliance, especially regarding the complex construct of subjective risk perception. Furthermore, this study analyzes data from the early phases of the pandemic, offering a good starting point to analyze learning effects and changing trends in compliance.

However, this study presents some limitations. First, the outcomes considered were self-reported frequencies regarding compliance with regulations, which might be prone to social desirability and recall bias More precise questions (for example “How often did you wear a mask *that fully covered your mouth and nose?*”) including more objective frequency categories would contribute to a more reliable measurement of compliance. Furthermore, the derived scales are not validated and suffer from a relatively low reliability (measured with Cronbach´s alpha). Second, the baseline sample might have suffered from selection bias, given that the questionnaire was available only in the German language. Furthermore, data was collected based on a door-to-door methodology during social distancing times, where medical personnel was often accompanied by the police. This might have helped to increase participation in some households, but might also have scared off more risk-averse and anxious individuals. Furthermore, the survey was designed to gather only essential data in order to increase the cooperation of individuals and, thus, response rates and returns to follow-up. However, this forced us to collect information only on a selection of potential correlates of compliance, leaving probably important factors out of the analysis as omitted/unobserved factors (such as time preferences, personality traits, trust in government/science/healthcare system). Additionally, the loss to follow-up was also relatively high (43%), with younger individuals, males and individuals from neighborhoods with more than 5 apartments per building who were less likely to take part in the follow-up (Supplementary material 6). Third, the study aims at estimating associations between the compliance factors and the variables included. This approach provided interesting insights, but the investigated relations should be further analyzed in the context of causal analysis to be able to inform policymaking and to create effective targeted strategies to increase compliance.

## Conclusions

Despite a growing share of vaccinated individuals, compliance with NPIs remains a key issue for epidemic control under all conditions where herd immunity is not being achieved, for example because vaccination campaigns are insufficient, are proceeding slowly, are facing anti-vaccination movements, or are facing immune escape by new virus variants [67]. Our study showed that younger individuals, men and individuals who received a positive serologic test previously should be targeted by measures to improve compliance with regulations. Risk-aversion and self-efficacy also played an important role for compliance. Future research should focus on investigating the causal pathways behind the reported associations in order to inform new and targeted policies. Moreover, the role of socioeconomic characteristics and migration background remains unclear, so that research with larger and more diverse populations is needed to formulate more precisely targeted policy implications.

## Supplementary Information

**Additional file 1:** **Supplementary material 1.** Details on the determinants of compliance. **Supplementary material 2.** Exploratory and confirmatory factor analyses. **Supplementary material 3.** Full descriptive statistics and imputation diagnostics. **Supplementary material 4.** Analysis of single behaviors. **Supplementary material 5.** Sensitivity analysis results. **Supplementary material 6.** Loss to follow-up analysis. **Supplementary material 7.** Acknowledgements.

## Data Availability

The data that support the findings of this study are available from the KoCo19 collaboration group, but restrictions apply to the availability of these data, which were used under license for the current study, and so are not publicly available. Data are however available from the authors upon reasonable request and with permission of the KoCo19 collaboration group.”

## Abbreviations

COVID-19: Corona Virus Disease 19
KoCo19: COVID-19 cohort study Munich
NPI: Non-pharmaceutical measures
OR: Odds-ratio
SARS-CoV-2: Severe Acute Respiratory Syndrome Coronavirus 2
SES: Socio-economic status
WHO: World Health Organization
