# Supplementary material for: Socioeconomic and risk-related drivers of compliance with measures to prevent SARS-CoV-2 infection: evidence from the Munich-based KoCo19 study
Source: BMC Public Health. 2023 May 11;23:860. doi: 10.1186/s12889-023-15759-9 (PMC10173220; doi:10.1186/s12889-023-15759-9)
Supplement: Supplementary file 1 — Additional file 1: Supplementary material 1. Details on the determinants of compliance. Supplementary material 2. Exploratory and confirmatory factor analyses. Supplementary material 3. Full descriptive statistics and imputation diagnostics. Supplementary material 4. Analysis of single behaviors. Supplementary material 5. Sensitivity analysis results. Supplementary material 6. Loss to follow-up analysis. Supplementary material 7. Acknowledgements. [file 12889_2023_15759_MOESM1_ESM.pdf]

Socioeconomic and risk-related drivers of compliance with measures to prevent SARS-CoV-2 infection: evidence from the Munich-based KoCo19 study

Sara Pedron, Michael Laxy, Katja Radon, Ronan Le Gleut, Jessica Michelle Guggenbüehl Noller, Maximilian Nikolaus Diefenbach, Noemi Castelletti, Michael Hölscher, Reiner Leidl, Lars Schwettmann and the KoCo19 collaboration group

Supplementary material

Supplementary material 1: details on the determinants of compliance

Supplementary material 2: exploratory and confirmatory factor analyses

Supplementary material 3: full descriptive statistics and imputation diagnostics

Supplementary material 4: analysis of single behaviors

Supplementary material 5: sensitivity analysis results

Supplementary material 6: loss to follow-up analysis

Supplementary material 7: acknowledgements

Supplementary material 1: details on the determinants of compliance

The following socioeconomic determinants were included in the analysis:

- **Age** was measured based on date of birth and divided in categories (20-34, 35-49, 50-64, 65-79, 80+) (measured at baseline)
- **Sex** was self-reported (male or female) (measured at baseline)
- **Education level:** the highest educational qualification was collected. Based on this information, the number of school years was computed. We then divided this variable in low education (<12 years of school), high education (>=12 years of school ~ upper secondary education diploma) and in school; (measured at baseline)
- **Employment status**: individuals were asked to choose their current employment status from a list (not employed (retired, in school, in university), unemployed, vocational training, employed, worker, self-employed, helping family members (unpaid), civil servant, soldier, gap year, side job, reduced working hours, no information) (measured at follow-up). For the analysis, we grouped them as follows:
    - Employed: employed, worker, civil servant
    - Self-employed: self-employed
    - Retired: not employed (retired, in school, in university) & age group>=50
    - Not employed/Other: not employed (retired, in school, in university) & age group<50, unemployed, vocational training, helping family members (unpaid), soldier, gap year, side job, reduced working hours
- Being in **risky employment** was assessed by asking individuals if they worked in risky occupations (measured at baseline). The list of possible answers was then divided as follows:
    - Health workers: retirement home, hospital, medical practice;
    - Other risky employment: sales employee (e.g. supermarket, etc), public transportation employee, air traffic employee, civil protection, other job with several contacts with the public.
- **Income** was measured as monthly net household income. The original values were then divided in categories: <=2500 €, 2500-4000 €, 4000-6000 €, 6000+ € (measured at baseline).
- **Migration background** was measured by asking individuals where they were born. The corresponding variable indicates thus if individuals were born in or outside Germany as a proxy for a direct migration background (measured at baseline).
- **Housing type**: study personnel retrieved information on how many apartments per building could be found in the area where the household was selected during the door to door visits. Possible answers were: 1-3 apartments per building, 3-4 apartments per building, 5 or more apartments per building (measured at baseline).
- **Living area per inhabitant** was computed by asking the head of the household how big was their apartment (in square meters) (measured at baseline).
- **Living with a partner** (measured at baseline).
- **Number of children in the household** (measured at baseline).
- **Chronic conditions**: individuals were asked if they have one chronic condition among the following: Diabetes (type 1 or 2), chronic lung disease, heart- or cardiovascular disease, cancer, obesity, allergy of the skin, allergy of the respiratory system, autoimmune disease, HIV infection, other chronic disease (measured at baseline). In the presence of one or more of these conditions, we defined the variable "chronic conditions" as "Yes", if none of these conditions was present, we defined the variable as "No".
- **Positive serologic test at baseline**: at baseline the study personnel retrieved blood samples of all participating household members, in order to run serologic tests to analyze the spread of the virus in the population (Radon et al., 2020). The results of these tests were communicated to participants within few weeks after participation to the baseline questionnaire.

- **Season**: this variable was coded summer or fall, depending on the day when the reply was received in the study center.

Descriptive statistics for all variables can be found in Table 1 and Table A.10.

Supplementary material 2: exploratory and confirmatory factor analysis for the scores

Risk perception score

The risk perception score was constructed based on three variables asking to which extent individuals perceived the risk of illness close, quickly spreading and threatening. Potential answers ranged between 1 (“far away”, “slowly spreading” or “not at all worrisome”) and 5 (“very near”, “quickly spreading” or “very worrisome”). Descriptive statistics are available in Table 1.

Table A.1: correlation.

	Risk (near)	Risk (quick)	Risk (threat)
**Risk (near)**	1		
**Risk (quick)**	0.35	1	
**Risk (threat)**	0.48	0.47	1

Table A.2: factor scores.

Variable	Factor1	Uniqueness
**Risk (near)**	0.7687	0.4090
**Risk (quick)**	0.7615	0.4201
**Risk (threat)**	0.8373	0.2990

The resulting score showed a Chronbach´s alpha of 0.70.

Figure A.3: distribution of risk perception score.

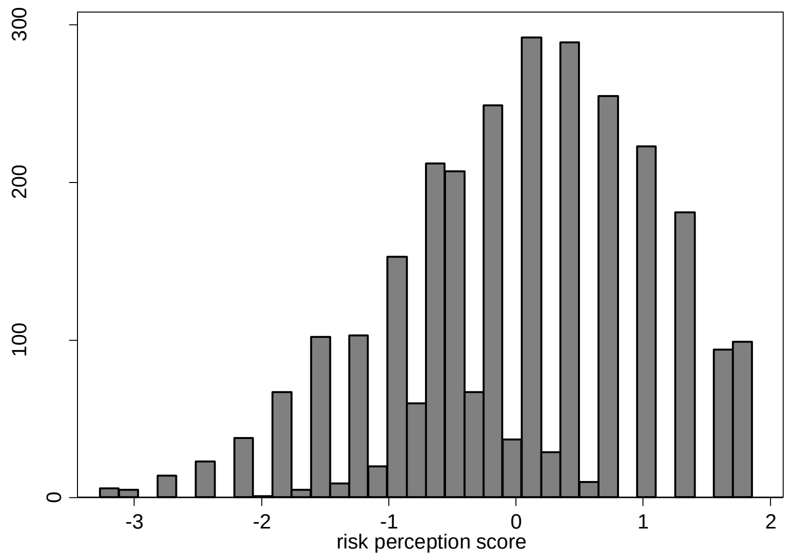

Worry score

The worry score was constructed based on our variables asking to which extent participants were worried about losing someone close, their own job and economic situation, and the whole economy. Potential answers ranged from 1 ("not worried at all") to 5 ("very worried"). Descriptive statistics are available in Table A.x.

Table A.4: correlation.

	Worry (lose someone)	Worry (own economic situation)	Worry (economy)	Worry (job)
**Worry (lose someone)**	1			
**Worry (own economic situation)**	0.30	1		
**Worry (economy)**	0.22	0.73	1	
**Worry (job)**	0.20	0.40	0.34	1

Table A.5: factor scores.

	Factor 1	Uniqueness
**Worry (lose someone)**	0.4820	0.7677
**Worry (own economic situation)**	0.8948	0.1993
**Worry (economy)**	0.8388	0.2965
**Worry (job)**	0.6660	0.5565

The resulting score showed a Chronbach´s alpha of 0.69.

Figure A.6: distribution of worry score.

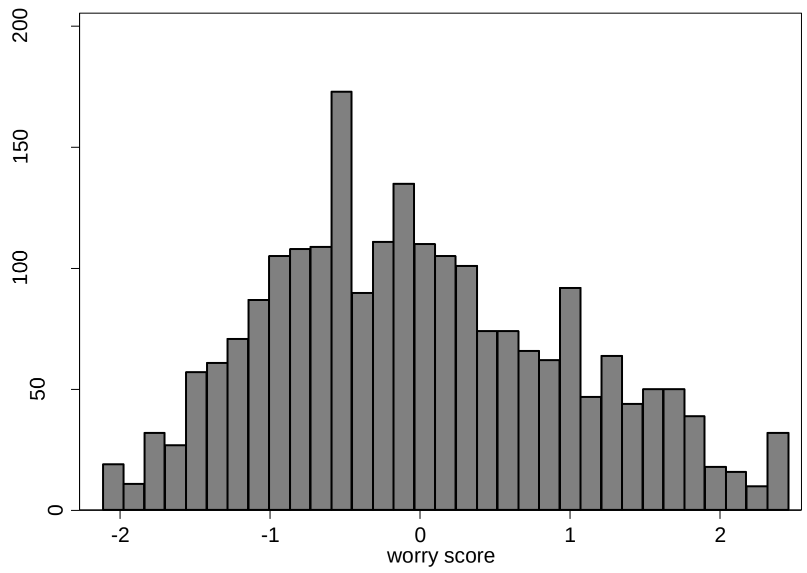

Compliance scores (Personal hygiene & social distancing)

Table A.7: correlation of the single dimensions of compliance

	Washing hands	Avoiding touching face	Wearing mask	Keeping distance	Avoiding social events	Avoiding public spaces
**Washing hands**	1					
**Avoiding touching face**	0.43	1				
**Wearing mask**	0.29	0.29	1			
**Keeping distance**	0.27	0.27	0.23	1		
**Avoiding social events**	0.15	0.15	0.15	0.21	1	
**Avoiding public spaces**	0.21	0.25	0.23	0.37	0.38	1

Fig. A.8: "personal hygiene" factor score distribution

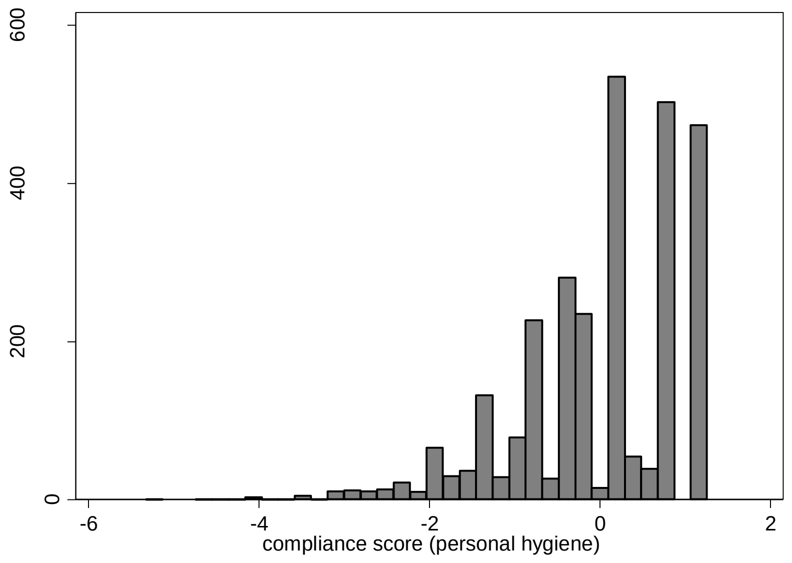

Fig. A.9: "social distancing" factor score distribution

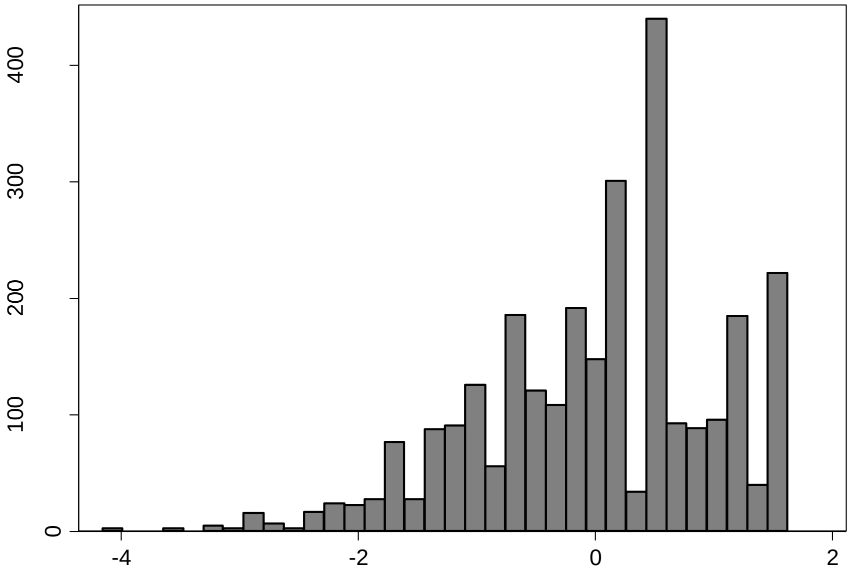

Supplementary material 3: full descriptives and imputation diagnostics

Table A.10: descriptive statistics (original and imputed values)

	original data				imputed data			
	total	N	freq.	mean (SD)	total	N	freq.	mean (SD)
**Sociodemographic**								
Age group	2880				2880			
20-34		636	22%			636	22%	
35-49		838	29%			838	29%	
50-64		852	30%			852	30%	
65-79		440	15%			440	15%	
80+		114	4%			114	4%	
Sex	2880				2880			
Female		1567	54%			1567	54%	
Male		1313	46%			1313	46%	
Education Level	2727				2880			
low (<12 years of school)		859	31%			910	33%	
high (>=12 years of school)		1868	69%			1970	72%	
Employment status	2870				2880			
Employed		1727	60%			1730	60%	
Self-employed		264	9%			265	9%	
Retired		587	20%			592	21%	
Not employed		292	10%			293	10%	
In risky employment (health)	2326				2880			
Yes		171	7%			203	9%	
No		2155	93%			2677	115%	
In risky employment (other)	2326				2880			
Yes		352	15%			429	18%	
No		1974	85%			2451	105%	
Monthly HH income (€)	2190				2880			
<=2500		356	16%			488	22%	
2500-4000		497	23%			644	29%	
4000-6000		711	32%			951	43%	
6000+		626	29%			797	36%	
Housing type	2880				2880			
1-4 apartments		983	34%			983	34%	
>=5 apartments		1897	66%			1897	66%	
Living area per individual	2795				2880			
<= 40sqm/individual		1558	56%			1604	57%	
>40sqm/individual		1237	44%			1276	46%	
Living with partner	2575				2880			
Yes		2101	82%			2363	92%	
No		474	18%			517	20%	
Number of children	2574			0.61 (0.95)	2880			0.63 (0.96)
Born outside Germany	2865				2880			
Yes		432	15%			434	15%	
No		2433	85%			2446	85%	
**Health status**								
Any chronic illness	2814				2880			
Yes		1202	43%			1240	43%	
No		1612	57%			1640	57%	
Positive serologic test at BL	2879				2880			
Yes		46	2%			47	2%	
No		2833	98%			2833	98%	
**Actual infection risk**								
7 day incidence/100.000 inhabitants	2880				2880			
0-35		2495	87%			2525	88%	
35-50		203	7%			173	6%	
>50		182	6%			182	6%	

Continues on next page.

Table A.11: descriptive statistics (original and imputed values) - *continued*

	original data				imputed data			
	**total**	**N**	**freq.**	**mean (SD)**	**total**	**N**	**freq.**	**mean (SD)**
**Risk perception**								
Subjective infection risk	2872			3.42 (1.26)	2880			3.42 (1.26)
Subjective infection severity	2865			4.18 (1.42)	2880			4.19 (1.42)
Level of information	2877			5.09 (1.16)	2880			5.09 (1.16)
(Score: risk perception)	2850			0.00 (1.00)	2880			0.00 (1.00)
Risk (near)	2864			4.56 (1.42)	2864			4.56 (1.42)
Risk (quick)	2871			4.74 (1.53)	2871			4.74 (1.53)
Risk (threat)	2870			5.16 (1.49)	2870			5.16 (1.49)
(Score: worry)	2250			0.00 (1.00)	2880			0.00 (0.99)
Worry (lose someone)	2873			4.06 (1.87)	2873			4.06 (1.87)
Worry (own economic situation)	2872			3.14 (1.82)	2872			3.14 (1.82)
Worry (economy)	2870			5.10 (1.50)	2870			5.10 (1.50)
Worry (job)	2258			2.75 (1.87)	2258			2.75 (1.87)
Risk aversion	2876			7.18 (2.10)	2880			7.18 (2.10)
Self-efficacy	2871			4.63 (1.63)	2880			4.64 (1.63)
**Season**	2880				2880			
Summer		2664	93%			2664	93%	
Fall		216	8%			216	8%	

Notes: summaries from the first imputation, only variables with missing data were imputed; the factor scores, not the original values, were imputed for risk perception and worry scores. For more information, see Supplementary material XX & XX.
BL=baseline.

Table A.12: missing values in original variables

	Complete	Incomplete	Imputed	Total
**Low education (<12 years)**	2727	153	153	2880
**Employment Status**	2870	10	10	2880
**Risky employment (Health)**	2326	554	554	2880
**Risky employment (Other)**	2326	554	554	2880
**Living with partner**	2575	305	305	2880
**Number of children**	2574	306	306	2880
**Monthly HH income**	2190	690	690	2880
**Living area**	2795	85	85	2880
**Born outside Germany**	2865	15	15	2880
**Any chronic illness**	2814	66	66	2880
**Positive serologic test (BL)**	2879	1	1	2880
**Subjective infection risk**	2872	8	8	2880
**Level of information**	2877	3	3	2880
**Subjective infection severity**	2865	15	15	2880
**Risk aversion**	2876	4	4	2880
**Self-efficacy**	2871	9	9	2880
**Risk perception (score)**	2850	30	30	2880
**Worry (score)**	2250	630	630	2880

The variables were imputed using predictive mean matching with 30 replications, using also age, sex, housing type, actual risk and month as additional covariates.

Supplementary material 4: analysis of single behaviors

Table A.13: analysis results (single behaviors)

	Washing hands			Avoiding touching face			Wearing a mask			Keeping a 1.5 m distance			Avoiding social gatherings			Avoiding public spaces		
	OR	95% CI	sig#	OR	95% CI	sig#	OR	95% CI	sig#	OR	95% CI	sig#	OR	95% CI	sig#	OR	95% CI	sig#
Sociodemographic																		
Age	1.15	[1.03; 1.28]		1.02	[0.93; 1.11]		0.72	[0.59; 0.88]	**	1.21	[1.07; 1.37]	*	1.10	[1.01; 1.2]		1.16	[1.07; 1.26]	**
Male	0.50	[0.41; 0.62]	***	0.63	[0.53; 0.74]	***	0.70	[0.48; 1.03]		0.84	[0.65; 1.08]		1.07	[0.91; 1.24]		0.94	[0.79; 1.1]	
Low education (vs. High)	1.36	[1.02; 1.83]		1.17	[0.93; 1.47]		1.38	[0.81; 2.37]		0.96	[0.69; 1.33]		0.91	[0.73; 1.12]		1.15	[0.92; 1.44]	
Employment Status (ref: employed)																		
Self Employed	1.34	[0.88; 2.05]		0.93	[0.68; 1.25]		0.93	[0.49; 1.75]		1.01	[0.62; 1.65]		0.91	[0.68; 1.22]		0.94	[0.7; 1.27]	
Retired	0.89	[0.56; 1.4]		0.98	[0.7; 1.38]		3.28	[1.41; 7.62]	*	1.42	[0.79; 2.53]		1.31	[0.95; 1.83]		1.08	[0.77; 1.52]	
Others - not employed	1.32	[0.9; 1.95]		1.15	[0.86; 1.55]		1.64	[0.75; 3.58]		0.82	[0.56; 1.2]		1.12	[0.85; 1.48]		1.21	[0.91; 1.61]	
In risky employment (health)	1.25	[0.76; 2.04]		1.26	[0.87; 1.82]		1.65	[0.59; 4.58]		1.00	[0.59; 1.7]		1.10	[0.77; 1.56]		0.82	[0.58; 1.14]	
In risky employment (other)	1.00	[0.72; 1.4]		0.79	[0.61; 1.03]		1.23	[0.65; 2.31]		0.92	[0.63; 1.33]		0.92	[0.72; 1.18]		0.71	[0.55; 0.93]	
Living with partner	0.79	[0.52; 1.18]		1.00	[0.73; 1.36]		0.88	[0.44; 1.78]		1.01	[0.63; 1.63]		0.84	[0.62; 1.13]		1.07	[0.79; 1.45]	
Number of children	0.98	[0.84; 1.13]		0.92	[0.81; 1.03]		0.89	[0.69; 1.15]		1.02	[0.87; 1.19]		1.14	[1.01; 1.28]		0.93	[0.83; 1.05]	
Monthy HH income (ref: <=2500 €)																		
2500-4000	0.96	[0.62; 1.5]		0.98	[0.71; 1.37]		1.93	[0.9; 4.14]		1.13	[0.71; 1.81]		0.80	[0.58; 1.1]		0.86	[0.62; 1.2]	
4000-6000	0.99	[0.63; 1.56]		1.09	[0.77; 1.54]		2.03	[0.94; 4.37]		1.32	[0.79; 2.18]		0.91	[0.66; 1.27]		0.91	[0.65; 1.29]	
6000+	1.15	[0.7; 1.89]		1.05	[0.72; 1.54]		2.24	[0.98; 5.1]		1.08	[0.63; 1.85]		0.89	[0.61; 1.28]		0.91	[0.62; 1.33]	
Housing type (>=5 apts)	1.07	[0.82; 1.4]		1.04	[0.84; 1.28]		1.07	[0.71; 1.63]		0.76	[0.56; 1.05]		1.24	[1; 1.52]		0.82	[0.67; 1.01]	
Living area (>40sqm/ind)	0.99	[0.73; 1.34]		0.92	[0.73; 1.15]		0.98	[0.59; 1.63]		1.20	[0.86; 1.66]		1.03	[0.82; 1.29]		1.01	[0.8; 1.27]	
Born outside Germany	1.03	[0.75; 1.42]		1.24	[0.96; 1.6]		0.90	[0.53; 1.53]		0.84	[0.6; 1.16]		1.05	[0.83; 1.33]		1.00	[0.79; 1.26]	
Health status																		
Any cronic illness	1.15	[0.9; 1.45]		1.17	[0.97; 1.42]		0.82	[0.55; 1.21]		1.10	[0.82; 1.47]		1.02	[0.85; 1.23]		0.96	[0.8; 1.15]	
Positive serologic test (BL)	0.37	[0.18; 0.75]	*	0.88	[0.43; 1.78]		0.24	[0.1; 0.59]	*	0.41	[0.2; 0.86]		0.65	[0.33; 1.27]		0.74	[0.42; 1.3]	
Actual infection risk (ref: 0-35)																		
>35-50	0.65	[0.42; 1]		0.98	[0.66; 1.45]		1.77	[0.63; 4.97]		0.97	[0.55; 1.72]		1.77	[1.2; 2.6]	*	1.07	[0.74; 1.55]	
>50	0.41	[0.15; 1.15]		1.55	[0.71; 3.38]		0.51	[0.2; 1.34]		0.67	[0.1; 4.75]		1.59	[0.57; 4.46]		1.71	[0.7; 4.21]	
Risk perception																		
Subjective infection risk	1.05	[0.93; 1.19]		0.97	[0.88; 1.07]		0.93	[0.76; 1.15]		0.86	[0.74; 1]		0.91	[0.83; 0.99]		0.84	[0.76; 0.92]	***
Level of information	1.36	[1.22; 1.51]	***	1.06	[0.97; 1.16]		1.15	[0.93; 1.42]		1.24	[1.1; 1.41]	**	1.06	[0.97; 1.16]		1.05	[0.96; 1.15]	
Subjective infection severity	1.18	[1.03; 1.35]		1.08	[0.98; 1.2]		1.11	[0.89; 1.39]		1.03	[0.87; 1.23]		1.10	[1; 1.22]		1.22	[1.1; 1.35]	***
Risk perception (score)	1.19	[1.06; 1.35]	*	1.27	[1.14; 1.4]	***	1.87	[1.5; 2.32]	***	1.45	[1.24; 1.7]	***	1.20	[1.08; 1.32]	***	1.36	[1.22; 1.5]	***
Worry (score)	1.02	[0.9; 1.16]		1.06	[0.96; 1.18]		1.21	[0.96; 1.52]		1.06	[0.91; 1.23]		1.02	[0.93; 1.13]		1.02	[0.92; 1.13]	
Risk aversion	1.43	[1.29; 1.58]	***	1.41	[1.29; 1.53]	***	1.48	[1.24; 1.76]	***	1.25	[1.11; 1.42]	***	1.34	[1.23; 1.46]	***	1.40	[1.29; 1.53]	***
Self-efficacy	1.15	[1.02; 1.29]		1.18	[1.08; 1.29]	***	1.14	[0.93; 1.39]		1.26	[1.1; 1.45]	**	1.06	[0.97; 1.16]		1.02	[0.93; 1.12]	
Season (Fall) (ref: Summer)	2.15	[0.81; 5.69]		0.89	[0.43; 1.83]		2.22	[0.88; 5.57]		1.76	[0.27; 11.73]		1.63	[0.61; 4.37]		1.18	[0.51; 2.69]	
Intercept	4.61	[2.41; 8.81]	***	2.69	[1.62; 4.44]	***	78.53	[25.72; 239.8]	***	4.69	[2.22; 9.9]	***	1.09	[0.66; 1.82]		1.26	[0.77; 2.06]	
N	2,872			2,874			2,870			2,873			2,868			2,870		

Notes: HH: household, BL:baseline, SD: standard deviation; Since we did not impute the outcome variables (very few missing values), the respective models only include a slightly reduced subset of observations. # corrected pval: significance of the p-value corrected using the Benjamini & Hochberg method. Significance: *p<.05, **p<.01, ***p<0.001.

Supplementary material 5: sensitivity analysis results

Table A.14: sensitivity analysis (compliant = always respecting the regulations)

	Washing hands		Avoiding touching face		Wearing a mask		Keeping a 1.5 m distance		Avoiding social gatherings		Avoiding public spaces	
	OR	95% CI	OR	95% CI	OR	95% CI	OR	95% CI	OR	95% CI	OR	95% CI
Sociodemographic												
Age	1.04	[0.96; 1.13]	1.08	[0.98; 1.18]	0.87	[0.8; 0.94]	1.13	[1.03; 1.24]	1.14	[1.05; 1.24]	1.13	[1; 1.27]
Male	0.53	[0.46; 0.62]	0.72	[0.6; 0.87]	0.61	[0.52; 0.72]	0.89	[0.75; 1.06]	0.95	[0.82; 1.1]	1.02	[0.81; 1.27]
Low education (vs. High)	1.29	[1.05; 1.59]	1.45	[1.14; 1.84]	1.36	[1.09; 1.7]	1.23	[0.98; 1.54]	0.87	[0.71; 1.07]	1.53	[1.16; 2.03]
Employment Status (ref: employed)												
Self Employed	0.79	[0.59; 1.07]	0.83	[0.57; 1.21]	0.96	[0.72; 1.28]	0.78	[0.55; 1.11]	1.05	[0.79; 1.39]	1.11	[0.73; 1.69]
Retired	1.36	[0.99; 1.85]	1.64	[1.17; 2.31]	2.75	[1.97; 3.85]	1.43	[1.04; 1.98]	1.64	[1.2; 2.24]	1.70	[1.17; 2.48]
Others - not employed	1.03	[0.77; 1.37]	1.28	[0.93; 1.76]	1.25	[0.94; 1.67]	1.20	[0.86; 1.65]	1.31	[1; 1.73]	1.22	[0.8; 1.86]
In risky employment (health)	1.12	[0.81; 1.57]	1.06	[0.7; 1.61]	0.99	[0.7; 1.4]	1.29	[0.89; 1.88]	0.97	[0.69; 1.36]	0.87	[0.5; 1.52]
In risky employment (other)	0.97	[0.75; 1.24]	1.02	[0.74; 1.41]	0.83	[0.64; 1.07]	1.07	[0.8; 1.45]	0.89	[0.69; 1.14]	0.84	[0.57; 1.24]
Living with partner	0.92	[0.69; 1.25]	0.81	[0.58; 1.13]	1.07	[0.78; 1.48]	0.95	[0.69; 1.29]	0.84	[0.63; 1.12]	1.00	[0.68; 1.49]
Number of children	1.04	[0.93; 1.16]	0.92	[0.8; 1.07]	1.04	[0.92; 1.17]	1.01	[0.89; 1.13]	1.08	[0.95; 1.21]	1.08	[0.9; 1.29]
Monthly HH income (ref: <=2500 €)												
2500-4000	0.86	[0.62; 1.2]	0.90	[0.63; 1.28]	0.94	[0.68; 1.29]	0.91	[0.64; 1.29]	0.91	[0.67; 1.25]	0.84	[0.56; 1.27]
4000-6000	0.75	[0.53; 1.06]	0.78	[0.53; 1.15]	0.96	[0.67; 1.36]	0.93	[0.65; 1.32]	0.89	[0.64; 1.23]	0.75	[0.5; 1.14]
6000+	0.88	[0.6; 1.28]	0.86	[0.56; 1.31]	0.81	[0.55; 1.18]	1.01	[0.69; 1.5]	0.80	[0.56; 1.16]	0.62	[0.38; 1]
Housing type (>=5 apts)	1.05	[0.86; 1.28]	1.04	[0.82; 1.32]	0.96	[0.77; 1.18]	0.77	[0.62; 0.95]	1.52	[1.23; 1.87]	1.10	[0.84; 1.46]
Living area (>40sqm/ind)	1.05	[0.84; 1.32]	0.60	[0.46; 0.79]	0.86	[0.69; 1.09]	0.85	[0.66; 1.08]	0.95	[0.75; 1.2]	0.96	[0.69; 1.32]
Born outside Germany	1.15	[0.91; 1.45]	1.96	[1.52; 2.53]	1.40	[1.09; 1.79]	1.45	[1.13; 1.86]	1.20	[0.96; 1.5]	1.20	[0.87; 1.66]
Health status												
Any cronic illness	1.15	[0.96; 1.37]	1.18	[0.95; 1.45]	1.23	[1.02; 1.49]	1.17	[0.96; 1.42]	1.02	[0.85; 1.22]	1.04	[0.8; 1.35]
Positive serologic test (BL)	0.75	[0.4; 1.42]	0.62	[0.26; 1.43]	0.46	[0.25; 0.83]	0.69	[0.34; 1.4]	0.72	[0.34; 1.51]	0.55	[0.24; 1.29]
Actual infection risk (ref: 0-35)												
>35-50	1.19	[0.84; 1.67]	1.08	[0.72; 1.62]	1.47	[1.02; 2.13]	1.48	[1.04; 2.12]	1.72	[1.2; 2.47]	2.34	[1.61; 3.41]
>50	0.96	[0.48; 1.91]	1.61	[0.77; 3.37]	1.83	[0.66; 5.08]	2.21	[1.08; 4.53]	3.48	[1.5; 8.09]	2.24	[0.99; 5.08]
Risk perception												
Subjective infection risk (SD)	0.92	[0.84; 1]	0.90	[0.8; 1.01]	0.92	[0.84; 1]	0.88	[0.8; 0.98]	0.96	[0.87; 1.05]	0.77	[0.67; 0.87]
Level of information (SD)	1.24	[1.13; 1.35]	1.17	[1.05; 1.3]	1.20	[1.1; 1.31]	1.12	[1.01; 1.23]	1.03	[0.94; 1.12]	1.06	[0.93; 1.21]
Subjective infection severity (SD)	1.16	[1.05; 1.28]	1.06	[0.94; 1.2]	1.09	[0.99; 1.2]	1.08	[0.97; 1.2]	1.04	[0.95; 1.15]	1.17	[1.02; 1.35]
Risk perception (score)	1.09	[0.99; 1.2]	1.14	[1.01; 1.29]	1.26	[1.14; 1.39]	1.14	[1.02; 1.28]	1.25	[1.13; 1.38]	1.30	[1.13; 1.49]
Worry (score)	1.09	[0.99; 1.2]	1.11	[0.99; 1.24]	1.08	[0.98; 1.2]	1.17	[1.04; 1.31]	0.94	[0.85; 1.03]	1.05	[0.9; 1.21]
Risk aversion (SD)	1.44	[1.31; 1.58]	1.35	[1.2; 1.52]	1.37	[1.26; 1.49]	1.24	[1.12; 1.37]	1.28	[1.18; 1.39]	1.24	[1.08; 1.41]
Self-efficacy (SD)	1.10	[1; 1.2]	1.04	[0.94; 1.15]	1.05	[0.96; 1.15]	1.15	[1.04; 1.26]	1.04	[0.95; 1.13]	0.96	[0.85; 1.08]
Season (Fall) (ref: Summer)	1.66	[0.88; 3.12]	1.49	[0.75; 2.99]	1.52	[0.59; 3.96]	1.30	[0.67; 2.53]	0.67	[0.31; 1.48]	2.17	[1; 4.68]
Intercept	0.77	[0.46; 1.27]	0.24	[0.13; 0.41]	3.04	[1.82; 5.07]	0.20	[0.12; 0.35]	0.33	[0.2; 0.53]	0.06	[0.03; 0.12]
N	2,872		2,874		2,870		2,873		2,868		2,870	

Notes: HH: household, BL:baseline, SD: standard deviation; Since we did not impute the outcome variables (very few missing values), the respective models only include a slightly reduced subset of observations.

Table A.15: sensitivity analysis (compliant = always, often, sometimes respecting the regulations)

	Washing hands		Avoiding touching face		Wearing a mask		Keeping a 1.5 m distance		Avoiding social gatherings		Avoiding public spaces	
	**OR**	**95% CI**	**OR**	**95% CI**	**OR**	**95% CI**	**OR**	**95% CI**	**OR**	**95% CI**	**OR**	**95% CI**
Sociodemographic												
**Age**	1.13	[0.94; 1.35]	1.08	[0.96; 1.21]	0.79	[0.56; 1.12]	1.54	[1.17; 2.03]	1.05	[0.96; 1.16]	1.14	[1.01; 1.28]
**Male**	0.43	[0.3; 0.62]	0.54	[0.43; 0.68]	0.55	[0.24; 1.25]	0.51	[0.29; 0.93]	1.08	[0.91; 1.29]	0.89	[0.72; 1.09]
**Low education (vs. High)**	1.47	[0.91; 2.38]	1.34	[0.96; 1.87]	2.32	[0.72; 7.5]	0.63	[0.32; 1.26]	0.94	[0.74; 1.2]	1.02	[0.75; 1.37]
**Employment Status (ref: employed)**												
**Self Employed**	1.43	[0.68; 3.01]	0.99	[0.66; 1.5]	0.95	[0.25; 3.65]	0.66	[0.25; 1.73]	0.97	[0.7; 1.33]	0.76	[0.51; 1.12]
**Retired**	1.16	[0.57; 2.38]	0.92	[0.58; 1.46]	4.91	[0.79; 30.64]	1.56	[0.34; 7.27]	1.14	[0.79; 1.64]	0.92	[0.57; 1.5]
**Others - not employed**	1.44	[0.76; 2.72]	1.24	[0.82; 1.9]	0.78	[0.2; 3.02]	0.61	[0.29; 1.31]	1.17	[0.84; 1.62]	0.79	[0.55; 1.13]
**In risky job (health)**	0.78	[0.37; 1.61]	0.88	[0.54; 1.43]	2.02	[0.23; 17.46]	0.55	[0.21; 1.43]	1.20	[0.78; 1.84]	0.83	[0.54; 1.28]
**In risky job (other)**	1.32	[0.72; 2.42]	1.01	[0.7; 1.45]	3.04	[0.45; 20.52]	0.86	[0.39; 1.86]	0.83	[0.63; 1.09]	0.77	[0.55; 1.09]
**Living with partner**	0.72	[0.36; 1.42]	0.80	[0.52; 1.22]	0.93	[0.25; 3.44]	1.69	[0.62; 4.55]	0.88	[0.63; 1.23]	1.51	[1.01; 2.24]
**Number of children**	0.95	[0.75; 1.19]	0.93	[0.8; 1.1]	1.51	[0.84; 2.73]	1.18	[0.78; 1.79]	1.14	[0.99; 1.3]	1.03	[0.88; 1.2]
**Monthly HH income (ref: ≤2500 €)**												
**2500- ≤4000**	0.96	[0.47; 1.94]	1.06	[0.68; 1.65]	1.27	[0.34; 4.69]	1.50	[0.57; 3.94]	0.87	[0.6; 1.26]	0.95	[0.62; 1.45]
**4000- ≤6000**	0.89	[0.45; 1.76]	1.47	[0.94; 2.29]	4.28	[0.72; 25.58]	1.19	[0.44; 3.21]	0.96	[0.66; 1.41]	0.82	[0.52; 1.3]
**>6000**	1.21	[0.56; 2.63]	1.28	[0.78; 2.1]	2.45	[0.48; 12.43]	0.76	[0.25; 2.29]	0.83	[0.56; 1.25]	0.76	[0.47; 1.24]
**Housing type (≥5 apts)**	0.95	[0.63; 1.45]	1.01	[0.75; 1.36]	1.36	[0.61; 3.04]	0.72	[0.33; 1.61]	1.13	[0.9; 1.43]	0.72	[0.53; 0.97]
**Living area (>40sqm/ind)**	0.92	[0.56; 1.49]	0.94	[0.69; 1.3]	1.55	[0.62; 3.84]	1.17	[0.53; 2.55]	1.10	[0.86; 1.42]	1.25	[0.9; 1.72]
**Born outside Germany**	1.16	[0.67; 2.01]	1.37	[0.96; 1.96]	2.34	[0.55; 10]	2.01	[0.7; 5.79]	1.12	[0.85; 1.47]	1.22	[0.89; 1.66]
Health status												
**Any chronic illness**	1.10	[0.76; 1.61]	1.05	[0.81; 1.36]	0.49	[0.21; 1.15]	0.74	[0.39; 1.39]	0.87	[0.71; 1.07]	1.14	[0.89; 1.46]
**Positive serologic test (BL)**	0.37	[0.15; 0.95]	0.52	[0.25; 1.09]	1.00	[1; 1]	0.37	[0.09; 1.49]	0.59	[0.28; 1.23]	0.43	[0.22; 0.85]
Actual infection risk (ref: 0-35)												
**>35-50**	0.32	[0.18; 0.56]	0.80	[0.5; 1.27]	0.49	[0.13; 1.9]	0.97	[0.23; 4.08]	1.56	[0.99; 2.45]	1.06	[0.65; 1.75]
**>50**	0.22	[0.06; 0.87]	0.95	[0.39; 2.32]	0.23	[0.05; 1.11]	0.09	[0.01; 1.49]	1.07	[0.32; 3.61]	2.90	[0.93; 9.06]
Risk perception												
**Subjective infection risk (SD)**	1.33	[1.08; 1.64]	1.04	[0.91; 1.19]	1.12	[0.72; 1.73]	0.98	[0.69; 1.4]	0.94	[0.85; 1.04]	0.91	[0.8; 1.03]
**Level of information (SD)**	1.32	[1.11; 1.58]	0.99	[0.87; 1.11]	0.84	[0.54; 1.32]	1.22	[0.88; 1.68]	1.10	[0.99; 1.21]	1.03	[0.92; 1.16]
**Subjective infection severity (SD)**	1.07	[0.84; 1.36]	1.13	[0.98; 1.3]	0.98	[0.65; 1.48]	1.10	[0.73; 1.64]	1.01	[0.9; 1.14]	1.05	[0.9; 1.21]
**Risk perception (score)**	1.25	[1; 1.55]	1.25	[1.08; 1.44]	2.48	[1.57; 3.94]	1.26	[0.84; 1.89]	1.19	[1.07; 1.33]	1.40	[1.21; 1.63]
**Worry (score)**	1.06	[0.85; 1.32]	1.04	[0.91; 1.19]	1.21	[0.74; 2]	0.98	[0.7; 1.38]	1.03	[0.92; 1.16]	1.05	[0.92; 1.19]
**Risk aversion (SD)**	1.53	[1.29; 1.81]	1.36	[1.21; 1.52]	1.34	[0.94; 1.93]	1.52	[1.16; 2]	1.34	[1.22; 1.47]	1.46	[1.3; 1.63]
**Self-efficacy (SD)**	1.03	[0.84; 1.27]	1.19	[1.05; 1.35]	1.14	[0.73; 1.79]	1.29	[0.94; 1.77]	1.03	[0.92; 1.14]	0.96	[0.84; 1.09]
**Season (Fall) (ref: Summer)**	3.42	[0.85; 13.87]	1.02	[0.45; 2.32]	2.14	[0.51; 8.98]	24.74	[0.89; 686.05]	2.91	[0.89; 9.5]	0.65	[0.24; 1.76]
**Intercept**	29.40	[10.23; 84.5]	7.32	[3.57; 15.01]	168.96	[19.49; 1464.9]	16.82	[3.11; 90.81]	2.61	[1.47; 4.63]	4.02	[2.04; 7.95]
N	2,872		2,874		2,870		2,873		2,868		2,870	

Notes: HH: household, BL:baseline, SD: standard deviation; Since we did not impute the outcome variables (very few missing values), the respective models only include a slightly reduced subset of observations.

Table A.16: sensitivity analysis (original values)

	Compliance - personal hygiene			Compliance - social distancing		
	OLS est	95% CI	pval	OLS est	95% CI	pval
Sociodemographic						
**Age**	-0.022	[-0.079; 0.035]		0.038	[-0.016; 0.091]	
**Male**	-0.257	[-0.357; -0.157]	***	-0.049	[-0.146; 0.049]	
**Low education (vs. High)**	0.024	[-0.108; 0.155]		0.020	[-0.106; 0.147]	
**Employment Status (ref: employed)**						
**Self Employed**	-0.114	[-0.301; 0.072]		-0.048	[-0.201; 0.105]	
**Retired**	0.295	[-0.014; 0.603]		0.061	[-0.203; 0.326]	
**Others - not employed**	0.247	[0.054; 0.441]	*	0.050	[-0.14; 0.239]	
**In risky job (health)**	0.159	[-0.03; 0.348]		-0.029	[-0.217; 0.159]	
**In risky job (other)**	0.037	[-0.099; 0.172]		-0.170	[-0.314; -0.026]	*
**Living with partner**	-0.106	[-0.316; 0.103]		0.021	[-0.181; 0.222]	
**Number of children**	-0.023	[-0.087; 0.041]		0.057	[-0.01; 0.124]	
**Monthly HH income (ref: ≤2500 €)**						
**2500- ≤4000**	0.020	[-0.208; 0.249]		0.067	[-0.147; 0.281]	
**4000- ≤6000**	0.061	[-0.172; 0.294]		-0.043	[-0.269; 0.182]	
**>6000**	0.121	[-0.129; 0.37]		-0.135	[-0.386; 0.116]	
**Housing type (≥5 apts)**	-0.103	[-0.226; 0.021]		-0.046	[-0.166; 0.075]	
**Living area (>40sqm/ind)**	-0.051	[-0.199; 0.096]		0.031	[-0.107; 0.17]	
**Born outside Germany**	0.198	[0.046; 0.349]	*	0.080	[-0.07; 0.231]	
Health status						
**Any chronic illness**	0.073	[-0.038; 0.183]		-0.020	[-0.132; 0.092]	
**Positive serologic test (BL)**	-0.398	[-0.941; 0.146]		-0.422	[-0.83; -0.015]	*
Actual infection risk (ref: 0-35)						
**>35-50**	-0.141	[-0.408; 0.125]		-0.017	[-0.244; 0.21]	
**>50**	0.153	[-0.377; 0.684]		0.448	[-0.18; 1.075]	
Risk perception						
**Subjective infection risk (SD)**	-0.017	[-0.075; 0.042]		-0.094	[-0.153; -0.035]	**
**Level of information (SD)**	0.064	[0.007; 0.12]	*	0.031	[-0.027; 0.088]	
**Subjective infection severity (SD)**	0.088	[0.023; 0.154]	**	0.080	[0.012; 0.147]	*
**Risk perception (score)**	0.175	[0.108; 0.242]	***	0.182	[0.114; 0.25]	***
**Worry (score)**	0.070	[0.013; 0.128]	*	0.066	[0.011; 0.122]	*
**Risk aversion (SD)**	0.267	[0.21; 0.324]	***	0.185	[0.128; 0.242]	***
**Self-efficacy (SD)**	0.042	[-0.015; 0.099]		0.085	[0.028; 0.141]	**
**Season (Fall) (ref: Summer)**	-0.153	[-0.651; 0.345]		-0.205	[-0.781; 0.372]	
**Intercept**	0.192	[-0.127; 0.511]		-0.160	[-0.486; 0.165]	
**R^2**	2858			2854		
**N**	0.202			0.204		

Notes: HH: household, BL:baseline, SD: standard deviation; Since we did not impute the outcome variables (very few missing values), the respective models only include a slightly reduced subset of observations.
Significance: *p<.05, **p<.01, ***p<0.001.

Table A.17: sensitivity analysis excluding positive cases at baseline

	Compliance - personal hygiene				Compliance - social distancing			
	OLS est	95% CI	pval	pval	OLS est	95% CI	pval	pval
Sociodemographic								
**Age**	-0.003	[-0.038; 0.032]	0.870		0.079	[0.043; 0.115]	0.000	***
**Male**	-0.325	[-0.391; -0.259]	0.000	***	-0.044	[-0.107; 0.018]	0.165	
**Low education (vs. High)**	0.157	[0.066; 0.247]	0.001	**	0.023	[-0.065; 0.112]	0.603	
**Employment Status (ref: employed)**								
**Self Employed**	-0.027	[-0.149; 0.095]	0.662		-0.048	[-0.173; 0.077]	0.454	
**Retired**	0.253	[0.118; 0.388]	0.000	***	0.157	[0.029; 0.284]	0.017	*
**Others - not employed**	0.117	[-0.001; 0.236]	0.053		0.056	[-0.069; 0.181]	0.380	
**In risky job (health)**	0.053	[-0.091; 0.197]	0.472		-0.001	[-0.146; 0.144]	0.986	
**In risky job (other)**	-0.040	[-0.142; 0.063]	0.449		-0.113	[-0.227; 0]	0.051	
**Living with partner**	-0.044	[-0.176; 0.089]	0.518		0.005	[-0.127; 0.137]	0.939	
**Number of children**	-0.013	[-0.061; 0.035]	0.594		0.024	[-0.025; 0.073]	0.335	
**Monthly HH income (ref: ≤2500 €)**								
**2500- ≤4000**	0.002	[-0.14; 0.145]	0.973		-0.051	[-0.195; 0.092]	0.481	
**4000- ≤6000**	-0.002	[-0.149; 0.145]	0.980		-0.030	[-0.176; 0.115]	0.685	
**>6000**	0.019	[-0.142; 0.18]	0.820		-0.079	[-0.243; 0.085]	0.346	
**Housing type (≥5 apts)**	-0.001	[-0.085; 0.084]	0.988		-0.030	[-0.115; 0.055]	0.489	
**Living area (>40sqm/ind)**	-0.070	[-0.168; 0.028]	0.164		0.009	[-0.09; 0.109]	0.857	
**Born outside Germany**	0.170	[0.068; 0.272]	0.001	**	0.080	[-0.023; 0.182]	0.128	
Health status								
**Any chronic illness**	0.068	[-0.007; 0.144]	0.076		0.003	[-0.073; 0.079]	0.942	
**Positive serologic test (BL)**	0.000	[0; 0]	0.000	***	0.000	[0; 0]	0.000	***
Actual infection risk (ref: 0-35)								
**>35-50**	-0.019	[-0.184; 0.147]	0.826		0.225	[0.073; 0.378]	0.004	**
**>50**	-0.001	[-0.274; 0.273]	0.996		0.355	[0.002; 0.709]	0.049	*
Risk perception								
**Subjective infection risk (SD)**	-0.011	[-0.051; 0.028]	0.578		-0.075	[-0.115; -0.035]	0.000	***
**Level of information (SD)**	0.092	[0.053; 0.131]	0.000	***	0.049	[0.011; 0.088]	0.012	*
**Subjective infection severity (SD)**	0.061	[0.019; 0.103]	0.004	**	0.056	[0.013; 0.099]	0.011	*
**Risk perception (score)**	0.122	[0.078; 0.165]	0.000	***	0.153	[0.108; 0.198]	0.000	***
**Worry (score)**	0.049	[0.006; 0.091]	0.026	*	0.033	[-0.01; 0.077]	0.131	
**Risk aversion (SD)**	0.209	[0.169; 0.249]	0.000	***	0.175	[0.136; 0.215]	0.000	***
**Self-efficacy (SD)**	0.062	[0.023; 0.101]	0.002	**	0.035	[-0.004; 0.074]	0.076	
**Season (Fall) (ref: Summer)**	0.229	[-0.022; 0.481]	0.073		0.172	[-0.163; 0.506]	0.314	
**Intercept**	0.058	[-0.158; 0.275]	0.598		-0.381	[-0.605; -0.156]	0.001	**
**R^2**	2858				2854			
**N**	0.202				0.204			

Notes: HH: household, BL:baseline, SD: standard deviation; Since we did not impute the outcome variables (very few missing values), the respective models only include a slightly reduced subset of observations.
Significance: *p<.05, **p<.01, ***p<0.001.

Table A.18: sensitivity analysis: seasonality vs. actual incidence rates (personal hygiene)

	Compliance - personal hygiene							
	Season				Actual infection risk			
	OLS est	95% CI	pval	pval	OLS est	95% CI	pval	pval
Sociodemographic								
**Age**	-0.001	[-0.036; 0.034]	0.949		-0.001	[-0.036; 0.034]	0.968	
**Male**	-0.327	[-0.392; -0.261]	0.000	***	-0.326	[-0.392; -0.26]	0.000	***
**Low education (vs. High)**	0.162	[0.072; 0.251]	0.000	***	0.164	[0.074; 0.254]	0.000	***
**Employment Status (ref: employed)**								
**Self Employed**	-0.030	[-0.151; 0.091]	0.626		-0.031	[-0.152; 0.091]	0.621	
**Retired**	0.240	[0.106; 0.374]	0.000	***	0.244	[0.111; 0.378]	0.000	***
**Others - not employed**	0.116	[-0.001; 0.234]	0.052		0.119	[0.001; 0.236]	0.048	*
**In risky job (health)**	0.049	[-0.093; 0.191]	0.499		0.046	[-0.096; 0.188]	0.529	
**In risky job (other)**	-0.031	[-0.133; 0.07]	0.547		-0.034	[-0.135; 0.067]	0.510	
**Living with partner**	-0.045	[-0.178; 0.087]	0.500		-0.043	[-0.175; 0.09]	0.527	
**Number of children**	-0.014	[-0.062; 0.034]	0.569		-0.014	[-0.063; 0.034]	0.563	
**Monthly HH income (ref: ≤2500 €)**								
**2500- ≤4000**	-0.001	[-0.142; 0.14]	0.987		-0.007	[-0.148; 0.134]	0.925	
**4000- ≤6000**	0.003	[-0.142; 0.149]	0.964		-0.001	[-0.148; 0.146]	0.991	
**>6000**	0.022	[-0.139; 0.182]	0.792		0.016	[-0.145; 0.177]	0.845	
**Housing type (≥5 apts)**	0.000	[-0.084; 0.084]	0.993		0.004	[-0.08; 0.088]	0.933	
**Living area (>40sqm/ind)**	-0.062	[-0.16; 0.036]	0.217		-0.063	[-0.161; 0.035]	0.209	
**Born outside Germany**	0.165	[0.064; 0.267]	0.001	**	0.167	[0.065; 0.268]	0.001	**
Health status								
**Any chronic illness**	0.063	[-0.012; 0.138]	0.098		0.065	[-0.01; 0.14]	0.092	
**Positive serologic test (BL)**	-0.407	[-0.716; -0.099]	0.010	*	-0.405	[-0.714; -0.097]	0.010	*
Actual infection risk (ref: 0-35)								
**>35-50**					0.000	[0; 0]	0.000	***
**>50**					0.019	[-0.128; 0.165]	0.801	
Risk perception								
**Subjective infection risk (SD)**	-0.012	[-0.051; 0.027]	0.551		-0.011	[-0.05; 0.029]	0.593	
**Level of information (SD)**	0.096	[0.057; 0.135]	0.000	***	0.097	[0.058; 0.136]	0.000	***
**Subjective infection severity (SD)**	0.060	[0.018; 0.101]	0.005	**	0.060	[0.018; 0.101]	0.005	**
**Risk perception (score)**	0.130	[0.087; 0.173]	0.000	***	0.131	[0.088; 0.175]	0.000	***
**Worry (score)**	0.046	[0.003; 0.088]	0.034	*	0.046	[0.003; 0.088]	0.035	*
**Risk aversion (SD)**	0.209	[0.169; 0.249]	0.000	***	0.208	[0.168; 0.248]	0.000	***
**Self-efficacy (SD)**	0.062	[0.023; 0.1]	0.002	**	0.062	[0.023; 0.1]	0.002	**
**Season (Fall) (ref: Summer)**	0.244	[0.112; 0.376]	0.000	***				
**Intercept**	0.048	[-0.167; 0.263]	0.661		0.046	[-0.169; 0.262]	0.674	
**R^2**	2858				2854			
**N**	0.202				0.204			

Notes: HH: household, BL:baseline, SD: standard deviation; Since we did not impute the outcome variables (very few missing values), the respective models only include a slightly reduced subset of observations.
Significance: *p<.05, **p<.01, ***p<0.001.

Table A.19: sensitivity analysis: seasonality vs. actual incidence rates (social distancing)

	Compliance - social distancing							
	Season				Actual infection risk			
	OLS est	95% CI	pval	pval	OLS est	95% CI	pval	pval
Sociodemographic								
**Age**	0.083	[0.047; 0.119]	0.000	***	0.079	[0.043; 0.115]	0.000	***
**Male**	-0.044	[-0.106; 0.018]	0.164		-0.044	[-0.107; 0.018]	0.164	
**Low education (vs. High)**	0.030	[-0.058; 0.119]	0.498		0.030	[-0.058; 0.118]	0.508	
**Employment Status (ref: employed)**								
**Self Employed**	-0.064	[-0.188; 0.06]	0.313		-0.063	[-0.188; 0.062]	0.325	
**Retired**	0.159	[0.032; 0.287]	0.014	*	0.154	[0.027; 0.281]	0.017	*
**Others - not employed**	0.041	[-0.084; 0.165]	0.519		0.046	[-0.078; 0.17]	0.467	
**In risky job (health)**	0.001	[-0.143; 0.145]	0.984		-0.007	[-0.151; 0.136]	0.922	
**In risky job (other)**	-0.109	[-0.223; 0.006]	0.063		-0.113	[-0.227; 0.001]	0.052	
**Living with partner**	0.006	[-0.125; 0.137]	0.928		0.014	[-0.116; 0.145]	0.830	
**Number of children**	0.026	[-0.022; 0.074]	0.288		0.025	[-0.023; 0.073]	0.311	
**Monthly HH income (ref: ≤2500 €)**								
**2500- ≤4000**	-0.071	[-0.214; 0.072]	0.331		-0.068	[-0.211; 0.075]	0.353	
**4000- ≤6000**	-0.055	[-0.201; 0.09]	0.453		-0.047	[-0.193; 0.099]	0.526	
**>6000**	-0.101	[-0.264; 0.063]	0.226		-0.099	[-0.262; 0.065]	0.236	
**Housing type (≥5 apts)**	-0.032	[-0.116; 0.052]	0.457		-0.030	[-0.114; 0.055]	0.491	
**Living area (>40sqm/ind)**	0.012	[-0.087; 0.111]	0.816		0.015	[-0.083; 0.114]	0.760	
**Born outside Germany**	0.080	[-0.021; 0.182]	0.120		0.079	[-0.022; 0.181]	0.124	
Health status								
**Any chronic illness**	-0.005	[-0.08; 0.071]	0.903		0.002	[-0.073; 0.078]	0.956	
**Positive serologic test (BL)**	-0.386	[-0.661; -0.112]	0.006	**	-0.376	[-0.653; -0.099]	0.008	**
Actual infection risk (ref: 0-35)								
**>35-50**					0.000	[0; 0]	0.000	***
**>50**					0.246	[0.102; 0.39]	0.001	**
Risk perception								
**Subjective infection risk (SD)**	-0.075	[-0.114; -0.035]	0.000	***	-0.071	[-0.111; -0.032]	0.000	***
**Level of information (SD)**	0.048	[0.01; 0.086]	0.013	*	0.048	[0.01; 0.086]	0.013	*
**Subjective infection severity (SD)**	0.054	[0.011; 0.097]	0.015	*	0.054	[0.011; 0.097]	0.013	*
**Risk perception (score)**	0.157	[0.112; 0.202]	0.000	***	0.157	[0.113; 0.202]	0.000	***
**Worry (score)**	0.030	[-0.013; 0.073]	0.172		0.032	[-0.011; 0.074]	0.148	
**Risk aversion (SD)**	0.180	[0.14; 0.22]	0.000	***	0.179	[0.139; 0.218]	0.000	***
**Self-efficacy (SD)**	0.036	[-0.003; 0.075]	0.067		0.038	[-0.001; 0.076]	0.056	
**Season (Fall) (ref: Summer)**	0.501	[0.361; 0.641]	0.000	***				
**Intercept**	-0.365	[-0.588; -0.142]	0.001	**	-0.377	[-0.599; -0.154]	0.001	**
**R^2**	2858				2854			
**N**	0.202				0.204			

Notes: HH: household, BL:baseline, SD: standard deviation; Since we did not impute the outcome variables (very few missing values), the respective models only include a slightly reduced subset of observations.
Significance: *p<.05, **p<.01, ***p<0.001.

Supplementary material 6: loss to follow-up analysis

43% of individuals did not responded to the follow-up.

Outcome variable: dummy variable equal 1 if the person took part in the follow up, 0 otherwise.

Only variables that were available for (almost) al individuals were used in the follow-up analysis.

Table A.20: loss to follow-up analysis

	OR	95% CI
**Age**	1.20	[1.16; 1.24]
**Male**	0.80	[0.72; 0.9]
**Positive serologic test (BL)**	0.89	[0.58; 1.36]
**Housing type (3-4 apts)**	0.68	[0.53; 0.87]
**Housing type (>=5 apts)**	0.87	[0.76; 0.99]
**Intercept**	0.78	[0.63; 0.95]
**N**	5042	

The table shows that male and younger individuals are less likely to participate again in the follow-up. Furthermore, individuals with a positive serologic test at baseline are slightly less likely to participate in follow-up, albeit this coefficient is not significant (probably due to the low number of individuals with a positive serologic test at baseline). Furthermore, individuals who live in houses with 3-4 apartments or more than 5 apartments are less likely to participate in follow-up compared to individuals who live in houses with 1 or 2 apartments.

Supplementary material 7: acknowledgements

We acknowledge the work of other researchers who worked with the KoCo19 collaboration group but did not contribute directly to this study:

Emad Alamoudi, Jared Anderson, Valeria Baldassare, Maximilian Baumann, Marc Becker, Marieke Behlen, Jessica Beyerl, Rebecca Böhnlein, Anna Brauer, Vera Britz, Friedrich Caroli, Lorenzo Contento, Alina Czwienzek, Flora Deák, Emma Dech, Laura Dech, Jana Diekmannshemke, Anna Do, Gerhard Dobler, Jürgen Durner, Ute Eberle, Tabea M. Eser, Philine Falk, Volker Fingerle, Stefanie Fischer, Marius Gasser, Sonja Gauder, Leonard Gilberg, Kristina Gillig, Philipp Girl, Elias Golschan, Vitus Grauvogl, Celina Halfmann, Tim Haselwarter, Matthias Herrmann, Stefan Hillmann, Christian Hinske, Janna Hoefflin, Tim Hofberger, Michael Höfinger, Sacha Horn, Christian Janke, Ursula Kappl, Charlotte Kiani, Isabel Klugherz, Norah Kreider, Arne Kroidl, Magdalena Lang, Clemens Lang, Silvan Lange, Ekaterina Lapteva, Felix Lindner, Alexander Maczka, Alisa Markgraf, Paula Matcau, Rebecca Mayrhofer, Anna-Maria Mekota, Hannah Müller, Katharina Müller, Leonie Pattard, Claire Pleimelding, Konstantin Pusl, Elba Raimúndez, Julius Raschka, Jakob Reich, Raquel Rubio-Acero, Nicole Schäfer, Paul Schandelmaier, Lara Schneider, Sophie Schultz, Heidi Seibold, Paul Stapor, Jeni Tang, Sophie Thiesbrummel, Eva Thumser, Niklas Thur, Julian Ullrich, Julia Waibel, Julia Wolff, Pia Wullinger, Tobias Würfel, Patrick Wustrow, Houda Yaqine, Sabine Zange, Thomas Zimmermann, Lea Zuche
